# Novel instrumentation in Urology: Promoting progress

**DOI:** 10.4103/0970-1591.70571

**Published:** 2010

**Authors:** Manoj Monga

**Affiliations:** Director, Steven Streem Center for Endourology and Stone Disease, The Cleveland Clinic Foundation, Cleveland, USA. E-mail: endourol@yahoo.com

Urology is recognized as a progressive surgical field, populated by innovative and inquisitive minds driven to develop new approaches to traditional challenges, which lead to improved outcomes and decreased surgical morbidity. Indeed, urologists have been at the forefront in the development of endoscopic surgery, laparoscopic surgery, robotic surgery, neuromodulation, and tissue ablative therapy.

In this symposium, experts in these fields summarize the critical evolutions in the instrumentation and techniques of these novel surgical procedures, which have facilitated the development and implementation of progress. The challenges of costs that have traditionally hindered the wide-spread acceptance across the world are now under added scrutiny, as healthcare costs are the target of aggressive reform. The challenges of training become more acute, as the complexities and specializations of technique and instrumentation increase every day.

Across borders, we must focus on the training and cost, to ensure that these great strides that have been accomplished in minimally invasive urology remain in the forefront of urologic care and continue to push the envelope to improve, refine, and evaluate with a critical eye.

